# On the Relevance of Using Bayesian Belief Networks in Wireless Sensor Networks Situation Recognition

**DOI:** 10.3390/s101211001

**Published:** 2010-12-03

**Authors:** Antoine B. Bagula, Isaac Osunmakinde, Marco Zennaro

**Affiliations:** 1 Department of Computer Science, University of Cape Town, 7707 Cape Town, South Africa; 2 CSIR Modelling & Digital Sciences, Building 17A, Pretoria, South Africa; E-Mail: iosunmakinde@csir.co.za; 3 The Abdus Salam International Centre for Theretical Physics, Trieste, Italy; E-Mail: mzennaro@ictp.it

**Keywords:** wireless sensor networks, energy efficiency, situation awareness, situation recognition, probabilistic model

## Abstract

Achieving situation recognition in ubiquitous sensor networks (USNs) is an important issue that has been poorly addressed by both the research and practitioner communities. This paper describes some steps taken to address this issue by effecting USN middleware intelligence using an emerging situation awareness (ESA) technology. We propose a situation recognition framework where temporal probabilistic reasoning is used to derive and emerge situation awareness in ubiquitous sensor networks. Using data collected from an outdoor environment monitoring in the city of Cape Town, we illustrate the use of the ESA technology in terms of sensor system operating conditions and environmental situation recognition.

## 1. Introduction

The combination of wireless sensors with the RFID technology [1,2] is emerging as an important segment of the first mile connectivity of a next generation ubiquitous Internet where the information will be accessed not only *anywhere* and *anytime* but also by *anyone* and using *anything*. Derived from the Latin world *ubique* which denotes everywhere, *ubiquitous computing*—also called *pervasive computing* and often related to as *ambient intelligence*—is a post-desktop model of human-computer interaction which considers a thorough integration of the information processing into our daily life by engaging heterogeneous computational devices and systems simultaneously into objects that we manipulate. These objects and devices are used in our activities to deliver different services in a heterogeneous environment that involves a number of applications, protocols, operating systems, processors, and architectures. As opposed to the desktop paradigm that considers only a single device designed for a specialized purpose, ubiquitous computing is based on a multi-technology, multi-device and multi-protocol paradigm involving (1) different protocols such as WiFi, wireless LAN technology, 802.15.4, code-division and time-division multiple access wireless communication protocols (2) individual devices powered by different processors and technologies such as PDAs, smart phones, sensors, RFID tags and readers and laptops and (3) all these protocols and devices being embedded into different architectures such as centralized, distributed, peer-to-peer to meet different networking needs.

A common vision of a *ubiquitous sensor network (USN)* consists of sharing small, inexpensive, and robustly inter-networked processing devices which are generally embedded into distinctly common-place ends such as homes, markets, hospitals, streets, and workplaces and distributed at all scales throughout everyday life to achieve functions at different layers of a heterogeneous environment. USN deployments involve thousands of nodes sensing/reading their environment and sending the sensor readings to a sink node playing the role of base station connected to a more powerful computing device called gateway where information is processed locally or disseminated to remote processing locations where appropriate decisions are taken concerning the environment to be monitored. This process results in massive datasets that require appropriate processing to reveal hidden patterns used in situation recognition, prediction, reasoning and control and appropriate decision making. In the decision making process under uncertainty where variables are often more numerous and more difficult to measure and control, deterministic processes are static and more error prone than probabilistic approaches. For instance, the decisions of deterministic models are often a 1 (one) or a 0 (zero) whereas life is full of uncertainty. Probabilistic models can guide a decision making by ensuring for example that an event may occur with 70% certainty while in deterministic models, a good decision is judged by the outcome alone. In probabilistic models, the decision-maker is concerned not only with the outcome value but also with the amount of risk each decision carries. The probabilistic modelling approaches for analysis and decision making under uncertainty are gaining more popularity than deterministic methods in today’s world. For instance, Dynamic Bayesian Networks (DBNs) [3,4] are successfully used for speech recognition; a business analyst may use Bayesian networks to help understand the relationship of a sales ratio with a set of other variables. While probabilistic models are very smart in handling complete knowledge in ignorant and risky situations about complex domains of interest, deterministic models can only handle situations with complete knowledge. This means that deterministic processes are imperfect for complex and heterogeneous environments such as USNs.

Several references can be found in [5–7] that debate on the problem of situation awareness (SA) representations and applications. Burkolter *et al.* [5] mention that three training methods (Emphasis shift training, situation awareness training, and drill and practice) can be used to improve attention management skills in process control. They examined attention skills and process control performance in familiar and non-familiar situations over a retention interval of several weeks. They aimed to support novices learning a highly complex and demanding task by providing them with attention-management strategies in order to reduce their mental workload. SA training supported the diagnosis of novel system or non-familiar faults, which is more challenging. However, they recommend further detailed investigation on the effectiveness of SA training to improve attention management.

In [6], an ontology model is used to represent various scenarios of situation awareness including its expressiveness and demonstrate its extensibility to the core ontology. The ontology is used to annotate specific instances of a situation to accommodate various scenarios of the interaction with the end user. The ontological approach here is based on main representational structure, thereby lacking commonality of concepts used in the analysis of situation awareness processing. In [7], an open source information analysis and visualization which uses virtual globes to support the development of disaster event situation awareness is presented. Using a humanitarian disaster management, the key technology used for the research is the Context Discovery Application (CDA), which is a geovisual analytic environment, designed to integrate implicit geographic information with Google Earth. The final result is a map, which serves as a visual medium to support the development of situation awareness in humans. They recommend further work in any other dimension of interest to improve situation awareness of disaster management, which requires collaborative efforts. In view of the diverse work above, the approaches appear as a shared situation awareness, which could misdirect decision making to inappropriate aspects of the tasks. That is, building a single situation awareness model representing an entire time and presenting same information to all users is not only inefficient but highly confusing. The same piece of information from situations on Monday may have different meanings from situations perceived on other days or time periods. Hence, similarly to the domain of command and control, individual time periods or days require specific sets of situation awareness models, which could be interdependent.

This paper addresses the issue of situation management in emerging ubiquitous sensor networks. Building upon a common vision of USNs, we propose a USN management system and a situation recognition technology which achieves situation awareness using temporal probabilistic networks. Using examples from an outdoor environment monitoring in the city of Cape Town in South Africa, we illustrate the use of the proposed technology to emerge situation recognition in terms of sensor system operating conditions and environment sensor readings. The remainder of this paper is organized as follows. Section 2 describes the system architecture and the main features of our proposed prototype USN management system while Section 3 describes the situation recognition framework. Section 4 reports on the experimental results obtained from two outdoor sensing in Cape Town. Our conclusions and directions for future work are presented in Section 5.

## 2. The Ubiquitous Sensor Network System

As illustrated by Figure 1(a) from [1], different layers are used by a Ubiquitous Sensor Network (USN) to provide different services to different types of applications in a multi-technology, multi-devices and multi-protocol platform. These include (1) a sensor networking layer where sensor and RFID devices are placed into the environment to sense what is happening and report to sink nodes via USN-bridges (2) A USN access networking layer where USN-bridges and sink nodes are used as an access network for the first-mile connectivity of a Next Generation Network (NGN) gateways (3) a USN middleware, residing inside or outside the gateway, used as an interface between the NGN and the application layer and (4) different applications embodied into a USN applications layer to perform tasks related to logistics, structural health monitoring, agriculture control, disaster surveillance, military field surveillance, disaster/crisis management and many others.

### 2.1. The Situation Recognition System

**Figure 1.**
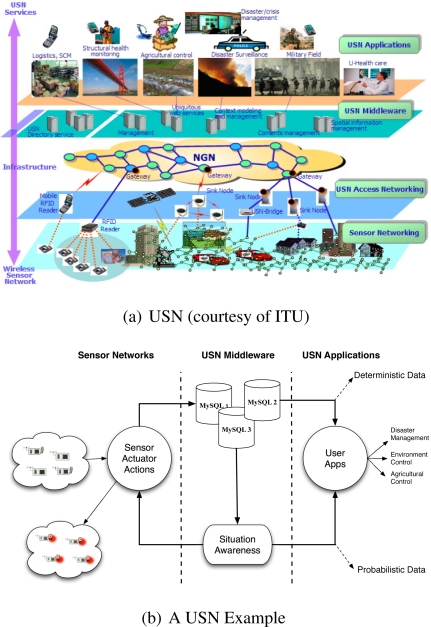
Ubiquitous Sensor Network. **(a)** USN Illustrated from [1]. **(b)** Situation Recognition System.

Building upon the layered approach depicted by Figure 1(a), we adopted a situation recognition system (SRS) depicted by Figure 1(b) where software and hardware components are used to deliver different services to the different layers of the system. The resulting SRS reveals (1) **sensors** and **actuators** which are deployed to sense what is happening into the environment (2) sensor readings stored into a set of **MySQL databases** to abstract the USN (3) a **situation awareness** component that uses the sensor readings which are stored into the MySQL databases and (4) a **user applications** interface that receives as input deterministic data and probabilistic data from the MySQL and ESA system respectively and ouput this data to different applications such as environment monitoring, agriculture control and disaster monitoring. While sensor and actuators are located into the sensor networks layer, the MySQL databases and the situation awareness are software components which are embedded into a gateway located in the USN middleware layer. The MySQL databases provide an interface to the application layer in terms of deterministic data and input to the situation awareness component. The situation awareness is another important component of the middleware layer that provides an interface to the application layer in terms of probabilistic data and the sensor networking layer in terms of reactive actions to control the environment. Note that while the USN architecture of Figure 1(a) reveals four layers, our SRS architecture includes only three layers since we have adopted a model that combines the USN access networking and USN middleware into a unique middleware layer where the USN is abstracted by the MySQL databases running into the gateways.

### 2.2. An Intelligent USN Middleware

**Figure 2.**
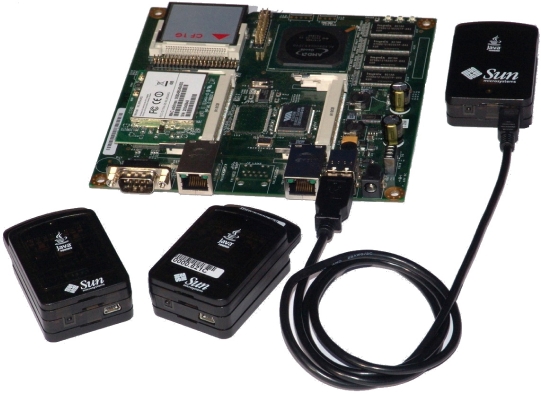
A Smart Board Connected to SUNSpot Devices.

While laptops and desktop systems may be used as gateways in USNs to host the middleware, we adopted a cheaper, flexible and more autonomous solution using smart boards such as the FoxBoard by ACME Systems [8] and the Alix board [9]. As depicted by Figure 2, the smart board hosting the wireless card and the CF memory card is connected via a USB cable to the SunSPOT [10] base station that communicates wirelessly with two SunSPOT motes. As described in [11], these smart boards are endowed with a rich set of interfaces to WiFi, Ethernet networks and with a USB and serial interfaces and are capable of using on-board database management systems such as MySQL with capabilities of automatic replication to MySQL enabled devices in a distributed network. We used a number of MySQL data base systems as the middleware layer providing an interface for layer 3 services and communicating with (1) the ESA module and (2) user applications module to improve the efficiency of the USN deployment. Residing between programs, operating systems, hardware and communication platforms of a heterogeneous environment to let the different applications and systems work together, the set of database management systems reside in the gateways to form the USN middleware. They are used as software components to abstract a USN [12] as datasets storing both the USN system operating parameters and sensor readings from the environment. The main functions achieved by the USN middleware consist of (1) hiding the underlying complexity of the environment (2) insulating the applications from explicit protocol handling, disjoint memories, data replication, network faults, and parallelism and (3) masking the heterogeneity of computer architectures, operating systems, programming languages, and networking technologies to facilitate application programming and management.

## 3. The Situation Recognition Framework

Building upon the SRS architecture depicted by Figure 1(b), we adopted an ESA system which can be useful for example in precision agriculture scenarios where (1) sensors are launched into a field to sense the humidity and temperature of the soil (2) the sensor readings are stored into a MySQL database that abstracts the USN (3) the MySQL databse is used as input to an ESA system that produces the conditional probabilities that the humidity levels are low and/or the light intensity is low and (4) reactive actions concerning the irrigation and/or switching the lights are taken based on these probabilities or the results of the ESA process are propagated as probabilistic data to be used by different other applications.

### 3.1. The ESA System Model

**Figure 3.**
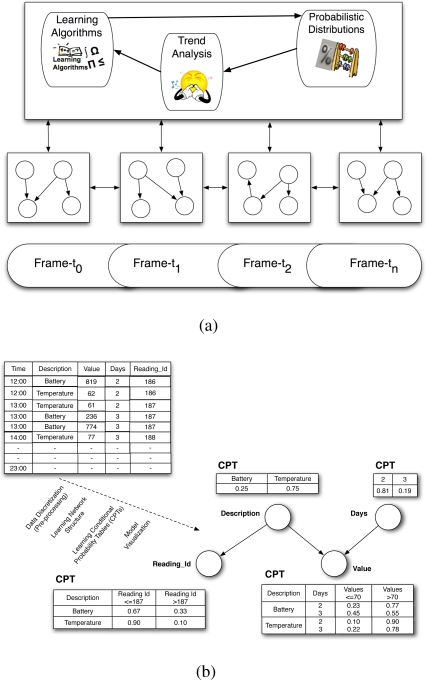
The Situation Recognition Framework. **(a)** The ESA system Model. **(b)** The Bayesian Learning Process.

The ESA extends the algorithms of ordinary Bayesian networks to truly emerge dynamically as it changes its network and the probabilistic distributions with time. As shown by the system model in Figure 3(a), it has three components: the learning algorithms, the probabilistic reasoner and the trend analyser. The learning component uses genetic algorithms [13] to emerge temporal Bayesian Network models, called frames, over the time steps from the multivariate time series (MTS) environments. This is illustrated in Figure 3(a) as the learning algorithms operate on the MTS with the objective of emerging the best or optimal temporal model for a time step which can change in subsequent time steps depending on situations. Emerging the best model for a time step requires an algorithm to iteratively do the following: (i) carry out a series of arc changes one at a time, (ii) check whether the resulting graph is a valid network structure, (iii) measure the network scores before and after every arc change, and (iv) replace the old structure with the new one depending on the difference between the scores. The probabilistic reasoner is the Bayesian inference engine, which handles the necessary possible forward and backward propagations through the links of the frames and generates probable results. The trend analyser is an interface engine that generates n-dimensional transition matrices of knowledge, where n corresponds to the pieces of hidden knowledge to be revealed, e.g., a transition matrix of target probabilities of situations, a transition matrix of target parameter values of events, *etc.* Examples of these parameter values are tabulated in Section 4.3 and the results of the ESA process are visualised in the figures of the same section.

### 3.2. The Baysesian Learning Process

As depicted by Figure 3(b), the sensor readings stored in a MySQL database system expressing the environment conditions such as temperature or humidity and/or the sensor system operating conditions such as the sensor battery levels are used as input to an intelligent middleware system to achieve situation awareness using situation recognition technologies such as the ESA [14]. The ESA is an innovative technology, which completely emerges temporal probabilistic models and reveals the hidden behaviour of what is currently happening over time in any domain of interest. One of its powerful features is its evolvement from multivariate time series (MTS) data in the absence of domain experts. The probabilistic models are Dynamic Bayesian Networks, which are often referred to as an extension of Bayesian Network (BN) models in artificial intelligence.

A Bayesian Network (BN) is formally defined as a directed acyclic graph (DAG) represented as G = {X(G), A(G)}, where X(G) = {*X*1*, . . ., Xn*}, vertices (variables) of the graph G and A(G) = {*A*1*, . . ., An*} is the set of arcs of G. The network requires discrete random values such that if there exists random variables *X*1*, . . ., Xn* with each having a set of some values *x*1*, . . ., xn* then, their joint probability density distribution can be defined such that a set of probabilistic parent(s) referred to as cause, has a dependency with a child variable known as effect. Every variable X with a combination of parent(s) values on the graph G captures probabilistic knowledge (distribution) as a conditional probability table (CPT).

The DBNs of the ESA are emerged over all the non-negative current time steps *t* ∈ *T*, such that T = {*t*1*, . . ., tn*} and the interlinked probabilistic relationships at each time step t is a Bayesian learning problem. As illustrated in Figure 3(b), learning such model dynamically from MTS can be decomposed as follows into sub-problems of (i) data discretization as a pre-processing step, (ii) learning the network structures over time, (iii) learning the associated CPTs (conditional probability tables) over time, and (iv) model visualization. Data discretization classifies numerical data into their corresponding interval values relatively to the patterns in the data attributes.

## 4. An Application to Outdoor Environment Monitoring

This section describes the experimental environment used to evaluate the performance of the proposed ESA framework and discusses the experimental results obtained in an outdoor setting when using a star-based single-hop wireless sensor network (WSN). The WSN was composed of five motes and a base station to sense temperature, humidity and light intensity in a sensing scenario where the sensor network was setup on top of the Groote Schuur hospital in Cape Town. Though many other aspects of the ESA framework are currently being investigated, the focus of this paper lies on the situation recognition in terms of environment situation awareness to find hidden patterns in the data captured about the environment and sensor system awareness using the link quality offered by sensor technology. While the former is expressed by the evolution of environment parameters such as light and temperature, the latter is based on the following sensor system performance parameters:

**Received Signal Strength Indicator (RSSI)**. This indicator was measured in dBm, and indicates the strength of the electromagnetic signal received by the base station, relative to a milliwatt. The RSSI in these experiments was measured by the base station operating in API mode. The minimum RSSI that the transmitting module is stated as being capable of receiving is -92 dBm; however RSSIs of -94 dBm were recorded during these trials.

**Packet Loss Indicator (PLI)**. The Packets Lost Indicator used is a percentage indication of the number of packets not received *versus* the total transmitted. The PLI used in these experiments was an absolute value, measured in terms of the unique identifier of each data packet from each Sensor, in the application layer. The unique identifier was implemented as a count, and missing data packets were measured by missing values in the count. The 802.14.5 MAC (the communication protocol used) specifies 3 retries if there is an acknowledgement failure from the receiver. As such, even if the packet is eventually received, this could have required several retransmissions. Evidence of these retransmissions could be sought in the relative Battery Lifetime Duration and RSSI of the sensors.

**Battery Life Duration (BLD)**. The Battery Lifetime was measured by comparing the time and date of the first and last transmission from each of the sensors. Additionally, the voltage across the battery was sampled and also transmitted with the other sensor data. This was used to check that the battery had in fact reached its minimum voltage before the sensor shut itself down. The battery life duration gives a good indication of the amount of retransmits required for each data packet, as cumulative additional retransmits significantly decrease battery lifetime.

### 4.1. Overview of Experiments

**Figure 4.**
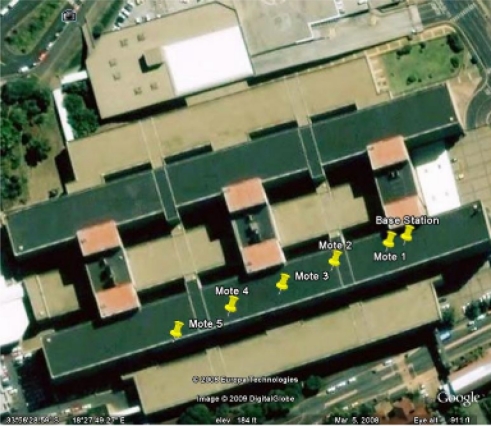
The Outdoor Testbed.

Two experiments were performed to evaluate the effect of distance on the performance of the motes. For the two experiments, the five motes were positioned at distances ranging from 10 m to 130 m from the base station. In the first experiment, all motes were set to transmit at full power, while in the second experiment all motes transmitted at a reduced power level. To ensure that the results of the experiment were as accurate as possible, a large open area was required that provided line-of-sight between all of the motes and the base station and that minimised the reflection of radio waves. Furthermore, the area needed to be as deserted as possible, as people walking about would interfere with the transmissions. At the end, the roof of Groote Schuur Hospital in Cape Town, South Africa was chosen as it matched the above criteria. Figure 4 shows the the test-bed, as well as the position of the base station and motes.

The distance of each mote from the base station, as well as each motes height above the ground, is shown in Table 1. As one can see from the table above, the further a mote was from the base station, the higher it was positioned above the roof surface. This test-bed location was used for the first two experiments. For all the experiments, the motes and base station were mounted on thin poles to raise them to the required heights. The motes and base station were also placed in plastic bags to protect them from the rain. Starting with a deterministic model, we conducted two experiments to evaluate the performance of our testbed by recording and graphing its main performance parameters in an USN scenario where a wireless sensor network was deployed on the roof of the Groote Schurr Hospital of the School of Medicine of the University of Cape Town. Thereafter, we used the ESA technology to emerge situation awareness for both the sensor system and the environment being controlled and reflect on its link to the deterministic model. The experiments were conducted for three days by having three sensor nodes located at different distances from the base station and using different output powers.

### 4.2. Applying a Deterministic Model

Using a deterministic model, we evaluated the performance of our sensor testbed by running the experiments at full and reduced power until the batteries in all the motes died (usually around 72 hours). This meant that each experiment replicated field conditions, as it was performed over an extended period of time, in varying weather conditions. However, during none of the experiments did it rain.

#### 4.2.1. Experiment 1: Distance Trial at Full Power

**Figure 5.**
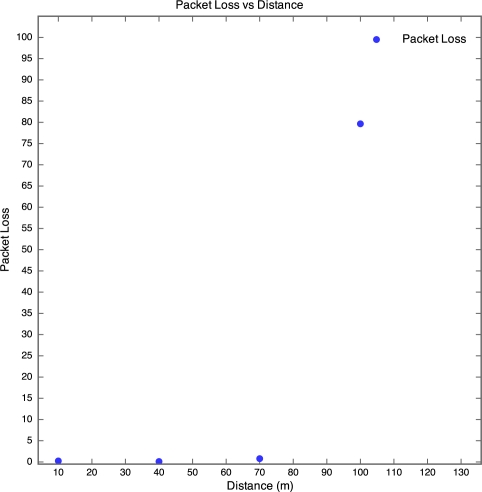
Packet Loss *vs.* Distance for Experiment 1.

**Figure 6.**
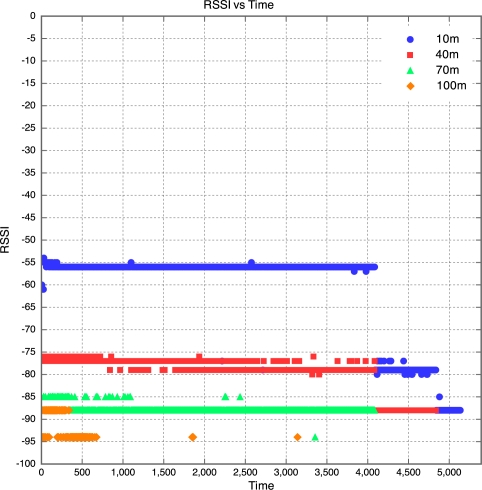
RSSI *vs.* Time for Experiment 1.

The aim of this experiment was to determine the effect of distance between motes on RSSI, PLI and battery life duration (BLD). Five motes were placed at distances of 10 m, 40 m, 70 m, 100 m and 130 m from the base station, as described earlier. All motes were configured to transmit at maximum power (0 dBm). It was found that the mote at 130 m was too far from the base station for any of the packets to get through. As a result, no packets were received from the 130 m base station. Packets were received from the node at 100 m, but there was considerable packet loss. This is shown in Figure 5 which clearly reveals that above 90 m, the packet loss approaches 100% at an exponential rate. Therefore, as long as the motes are kept less than 90 m apart, the packet loss can be kept below 10%, giving good performance. The RSSI for each mote is plotted against time in Figure 6. Each point on the graph indicates the RSSI of the packet at that time for that mote. Therefore, missing points indicate lost packets.

The RSSI quite clearly decreases with an increase in distance, as would be expected. The receiver in the XBee RF module is sensitive down to -92 dBm. The mote at 100 m is sitting at -94 dBm, which seems to be the limit. As a result, many packets from the 100 m mote were lost. This also explains why no packets were received from the 130 m mote, as the RSSI of the packets from this mote would have been less than the RSSI of the packets from the 100 m mote, meaning the RSSI would have been well below the receiver sensitivity threshold of -92 dBm. Although the RSSI for each mote was fairly constant over time, there does seem to be a slight downward trend in the RSSI plots for the motes. This explains why it was found that the PLR for each mote increased with time. This is illustrated in Figure 6, where one can see that the majority of the packet losses for the 100 m mote occurred only after the 10 hour mark.

**Figure 7.**
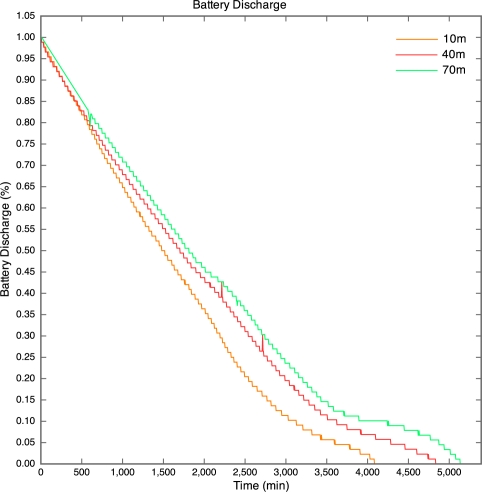
Battery Discharge Curves for Experiment 1.

The battery discharge curves for the motes are given in Figure 7. The discharge curve for the 100 m mote is incomplete, as all packets after the 43.5 hour point were lost. The discharge curves are non-linear due to the nature of lithium polymer batteries. The discharge curves have a similar shape to that of the typical discharge curve for the lithium polymer cells used in the motes.

**Figure 8.**
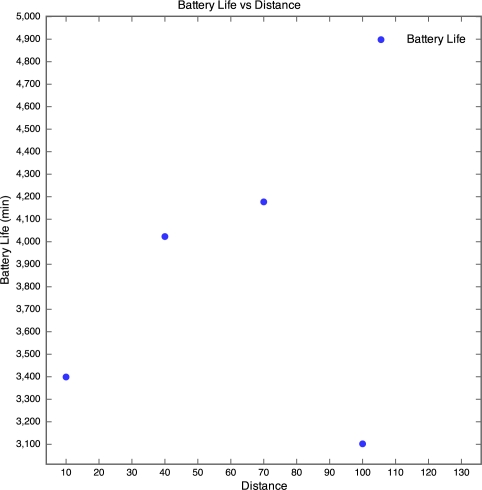
Battery Lifetime for Experiment 1.

The discharge curve is steeper for motes closer to the base station than those further away. The result of this is that the battery life was longer for motes further away than for those nearer to the base station as depicted by Figure 8.

In Figure 8, no plot was made for the 130 m mote, as the battery discharge curve was incomplete and there was no way of knowing when the mote actually died. However, the discharge curve of the 100 m mote does seem to follow the discharge curve of the 40 m mote. Therefore it seems realistic to estimate that the battery life of the 100 m mote would be similar to that of the 40 m mote, around 70 hours. Although no packets were received from the 130 m mote, the battery lifetime was determined manually by visually inspecting the mote on a regular basis and using the power and status LEDs as an indication of whether the mote was still running or if it had died. The normal behaviour of a WSN is for the furthest motes from the base station to have the shortest battery life as they perform more retransmissions than the closer motes. However, the motes seemed to exhibit the opposite behaviour. This is probably due to the fact that the time taken to wake a mote from sleep (0.5 seconds) is far longer than the time taken to retransmit a lost packet. Since the time taken to perform retransmissions is negligible and the mote draws a constant amount of current when awake, regardless of whether it is actually transmitting or not, an increase in distance would not cause a significant decrease in battery life. We were unable to explain though why the battery life increased with distance, instead of remaining constant.

#### 4.2.2. Experiment 2: Distance Trial at Reduced Power

**Figure 9.**
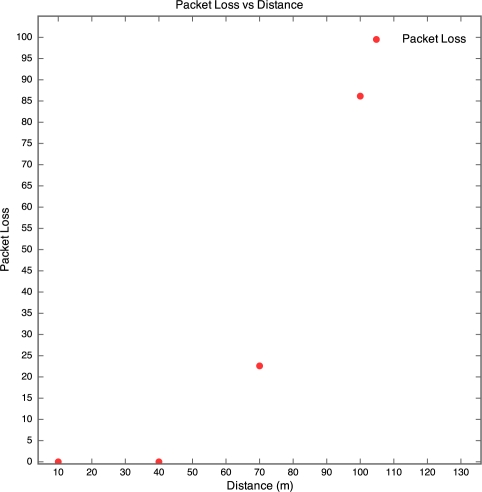
Packet Loss *vs.* Distance for Experiment 2.

Experiment 2 is a repeat of Experiment 1, with the motes now transmitting at the reduced power level of -4 dBm (as compared to 0 dBm in Experiment 1). At this reduced power level, we would expect to see higher packet losses, lower RSSI values and longer battery lifetimes than in Experiment 1. The packet losses for each mote are given in Figure 9. Note that no mote was placed at the 130 m point for this experiment, as we already knew from the previous experiment that all the packets from this mote would be lost.

**Figure 10.**
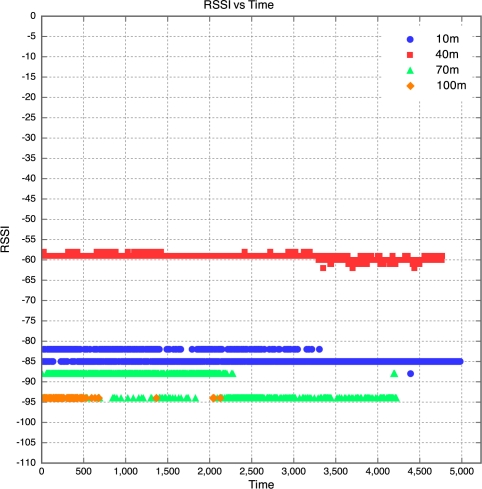
RSSI *vs.* Time for Experiment 2.

Again it can quite clearly be seen that packet loss increases with distance. The packet loss percentage for both the 70 m and 130 m motes increased significantly (0.8% to 23% and 80% to 86% respectively), when compared with Experiment 1. However, for the 10 m and 40 m motes, the packet loss did not increase at all when the transmit power was decreased. This indicates that if all the motes are less than 50 m apart, the transmit power can be decreased without increasing the packet losses. However, at this reduced power level, it seems that the motes do need to be within 60 m of each other to avoid excessive packet losses. Similarly, the RSSI for each mote decreased, although in many cases not by a significant amount. The exception was the 10 m mote which decreased from -56 dBm to -84 dBm. Strangely enough, the RSSI for the 40 m mote actually increased from -76 dBm to -59 dBm. This is because over such short distances interference from the environment has a bigger effect on the RSSI that signal attenuation. More importantly however, at the decreased transmit power level, neither the 10 m nor the 40 m mote lost any packets, and the RSSI only decreases slightly for the 70 m and 100 m motes as illustrated by Figure 10.

**Figure 11.**
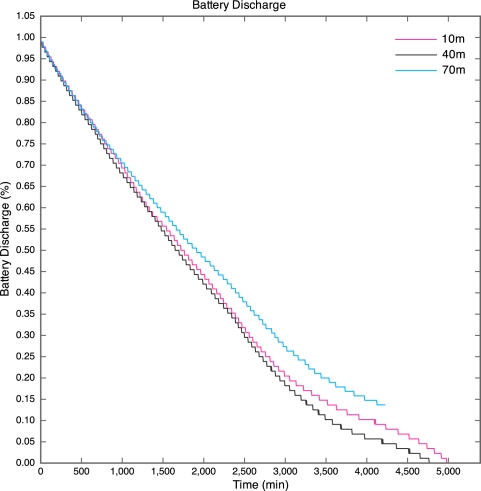
Battery Discharge Curves for Experiment 2.

**Figure 12.**
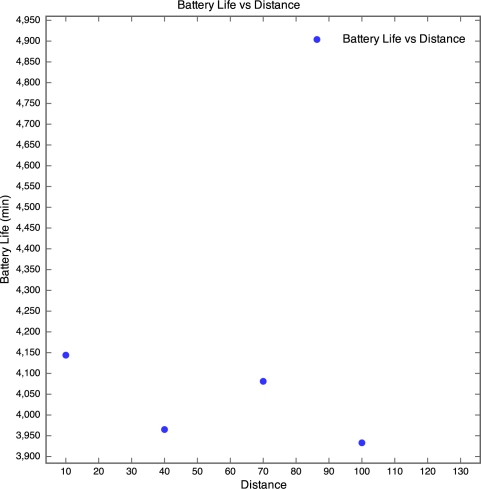
Battery Lifetime *vs.* Distance for Experiment 2.

The discharge curves for the batteries for the motes are given in Figure 11. The discharge curve for the 70 m mote requires some investigation. It was found that the battery pack for the 70 m mote was charged to a higher voltage than the other battery packs. Therefore, the discharge curve of the 70 m mote is flatter than the curves of the other motes not because it was discharging at a much slower rate than the other motes, but because its initial battery voltage was much higher (0.1 V higher). This also explains why the battery lifetime of the 70 m mote was much higher than the lifetime of the other motes, as depicted in Figure 12.

Just before the 60 hour mark, the pole mounting for the 70 m mote blew over. After this occurred, no more packets were received from the mote, which explains why the discharge curve stops just before 60 hours. However, based on the shape of the curve and data collected from other experiments, we estimated that the mote would have lasted 79 hours before the battery died. The discharge curve for the 100 m mote is incomplete as all packets after the 58.5 hour mark were lost, as the RSSI dropped too low. As a result, we were unable to determine the battery lifetime for the 100 m mote at the reduced transmit power.

Due to the fact that the 70 m motes battery was charged to a higher initial voltage than the batteries of the other motes and the fact that the discharge curve for the 100 m mote was incomplete, it is not possible to draw any definite conclusions regarding battery life of the motes from the second experiment.

### 4.3. Applying the Probabilistic Model

As part of its functionalities, our ESA system was designed to guide sensor managers through correct and precise decision making processes using an interactive functionality of question and answer session. For instance by applying the algorithms of the ESA, it captures say, sensor readings (e.g., battery, light, temperature, *etc.*), preprocesses them and emerges a network model over some space T of 24 hours time steps. Having obtained the model, one may reason and inquire to know for example the situation of battery values on any specific day over time. The predicted probabilities of the situation by the inference engine subsequently infer the most likely corresponding battery values.

**Figure 13.**
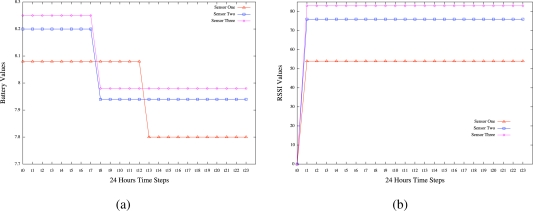
System Situation Awareness for day3: Scenario1. **(a)** Battery Life Awareness; **(b)** Signal Strength Awareness.

**Figure 14.**
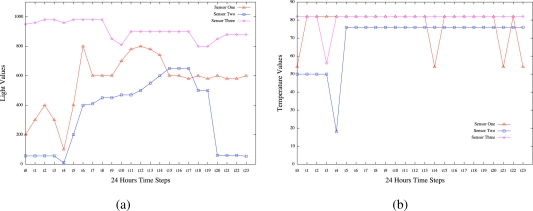
Environment Situation Awareness for Day3:Scenario1. **(a)** Light Intensity Awareness; **(b)** Temperature Awareness.

Using the ESA technology, we reconsidered the three days testbed experiments to emerge situation awareness in the same USN scenario where a wireless sensor network was deployed on the roof of the Groote Schurr Hospital of the School of Medicine of the University of Cape Town. Our objective was to detect hidden patterns in the data collected by the wireless sensor network in order to achieve sensor system awareness and environment situation awareness by finding *what is happening* and *why it is happening* to the environment and the sensors and *what can be done* to avoid unwanted behaviour. While simple interpretations can be used by expert people to achieve these findings, the advantage of using the emergent situation awareness technology is to lead less expert people to similar findings with less efforts. A probabilistic model is automatically emerged directly from the sensor readings by the learning algorithms of the ESA. The inference engine in turn acts on the model to provide collective intelligence results about any situations on the sensors over time. The results derived from the application of the ESA technology are depicted by Figure 13 for the sensor system awareness and Figure 14 for the environment situation awareness. Note that as depicted by the two figures, the battery and temperature measures are relative values that should be divided by a calibration parameter to reflect real voltage and temperature patterns.

The values presented in Table 2 express the degrees of beliefs (DoB) corresponding to a 24 hours time steps for one of the sensors (sensor one) starting from midnight (*t*_0_) to eleven in the night (*t*_23_). Similar probability patterns were found for the other sensors. They have not been reported for space limitiation. The experimental results depicted by the related figures reveal four situation recognitions:

- **Battery life awareness** revealed by Figure 13(a) with probabilities as degrees of beliefs (DoB) corresponding to the time steps, e.g., DoB for sensor one is computed by the ESA inference engine, which is based on Bayes’theorem, as set by column 1 in Table 2.
- **Signal strength awareness** revealed by Figure 13(b) with probabilities as degrees of beliefs (DoB) corresponding to the time steps, e.g., DoB for sensor one is computed by the ESA inference engine, which is based on Bayes’ theorem, as set by column 2 in Table 2.
- **Light intensity awareness** revealed by Figure 14(a) with probabilities as degrees of beliefs (DoB) corresponding to the time steps, e.g., DoB for sensor one is computed by the ESA inference engine, which is based on Bayes’ theorem, as set by column 3 in Table 2.
- **Temperature awareness** revealed by Figure 14(a) with probabilities as degrees of beliefs (DoB) corresponding to the time steps, e.g., DoB for sensor one is computed by the ESA inference engine, which is based on Bayes’ theorem, as set by column 4 in Table 2.

We note that the high degrees of beliefs show reliability on the environmental situation awareness. The combination of these figures and the associated probabilities provides answers to the probabilistic questions raised in the following subsections and evidences that will guide the trend analysis on the USN system considered in terms of questions and answers. These questions are referred to by *Q_i_* and the related answers are called *A_i_* where the *i* ∈ [1 . . . 4] indices represent the different questions.

#### 4.3.1. Experiment1: Sensor System Awareness

The functionalities of the system reveals the situational results and provides questions (or thinking approach) to be answered by the users towards a precise guide to correct decision making processes.

Figure 13(a) reveals the results obtained by a user when raising the following Probabilistic queries concerning the battery level of the sensors:

**Pr(Value t ?** ***|*** **Description t = Battery, Days t = 2) for all values of sensors 1, 2, and 3.**

Q1: What is Happening?

A1: The figure reveals that the battery voltages of the 3 sensors generally have similar behaviour but operate at varied power levels. It is observed that the power of sensor one operates uniformly at 8.08 V until it drops to 7.80 V at 12 pm. It uniformly remains at this level for the rest of its operation. One can see the other sensors operating similarly in the same way.

Q2: Why is it happening?

A2: Since most of the 3 sensors start dropping or change states at 7am, one could think that the power of the batteries are suspiciously affected by external interferences, environmental noise or high volume of workload. However, this seems to be a normal battery discharge pattern for the batteries since they were designed to be depleted after three days before recharge. The figure reveals also that sensor one is depleted quicker than the other two sensors. This is because this sensor was placed as a longer distance from the base station and configured to operate with higher output power in order to reach the base station.

Q3: What can we do about it?

A3: If environmental noise is a significant factor that may affect the power of the batteries, protective layers may be used as lagging to prevent or minimise the noise.

Q4: What will happen next?

A4: By Newton’s 1st law of motion, the power probably remains at these levels on this day until the next day when more workload or another impact of noise is received to further drop the voltage levels.

Figure 13(b) reveals the results obtained by a user when raising the following Probabilistic queries concerning the received signal strength (RSSI) of the sensors:

**Pr(Value t ?** ***|*** **Description t = RSSI, Days t = 2) for all values of sensors 1, 2, and 3.**

Q1: What is Happening?

A1: The RSSI of the 3 sensors are also generally similar in behaviour but operate at varied levels. Observe the RSSI of sensors 2 and 3 having close values 76 and 80 respectively, which remain uniformly constant over time.

Q2: Why is it happening?

A2: This might be due to the levels of operations of the sensors’ RSSI as they are directly proportional to the order of operations of the sensors batteries. One can see Figures 1 & 2 that sensor 3 level is the highest, followed by sensors 2 and 1. This is in agreement with what was revealed by answer A1 concerning sensor one being far from the base station as compared to the other two sensors.

Q3: What can we do about it?

A3: It is suspected that any possible actions taken on the sensors batteries will have effects on the RSSI levels of operations.

Q4: What will happen next?

A4: It is difficult to predict what will happen next in this case.

#### 4.3.2. Experiment2: Environment Situation Awareness

Figure 14(a) reveals the results obtained by a user when raising the following Probabilistic queries concerning the light intensity revealed by the sensors:

**Pr(Value t ?** ***|*** **Description t = Light, Days t = 2) for all values of sensors 1, 2, and 3.**

Q1: What is Happening?

A1: The signals of light sent by the sensors to the gateway have similar patterns as one can see that they are greatly based on day and night. Between 4-5 am, the intensity of light increases and they start dropping around 7-8 pm.

Q2: Why is it happening?

A2: The longer day and shorter night of summer periods are reflected in the situational patterns revealed.

Q3: What can we do about it?

A3: To send high light signals in the night may require the provision of artificially generated light in the sensors environment.

Q4: What will happen next?

A4: If the next day is still summer time, then similar situational patterns are likely revealed.

Figure 14(b) reveals the results obtained by a user when raising the following Probabilistic queries concerning the temperature revealed by the sensors:

**Pr(Value t ?** ***|*** **Description t = Temperature, Days t = 2) for all values of sensors 1, 2, and 3.**

Q1: What is Happening?

A1: Most of the temperature signals sent by the 3 sensors are above the level 70. They have very few irregular drops.

Q2: Why is it happening?

A2: The temperature is as high as that because summer is associated with hot environment. The irregular drops in temperature might result from the Cape strong winds.

Q3: What can we do about it?

A3: If the experiment is repeated in winter, then the temperature falls and the irregularities may be controlled.

Q4: What will happen next?

A4: If the experiment is repeated in summer, similar patterns are likely to be revealed because summer has fixed properties.

## 5. Conclusions and Future Work

Building upon a common vision of pervasive computing, this paper presents a network management system for ubiquitous sensor networks and proposes a framework using the ESA technology to achieve situation recognition. Using an outdoor environment monitoring in the city of Cape Town, we illustrate the use of the proposed technology in terms of sensor system operating conditions and environmental situation awareness. There is room to extend the situation recognition framework proposed in this paper to achieve future situation awareness when predicting future situations from current datasets. The scalability of the ESA technology when dealing with the massive datasets collected from sensor readings is another issue that needs to be addressed for the wide deployment of this technology in ubiquitous sensor networks. It is another avenue for future research work.

## Figures and Tables

**Table 1.** Experimental Setting.

Mote	Distance from base station	Height above roof

base station	-	0.36 m
Mote 1	10 m	0.36 m
Mote 2	40 m	0.6 m
Mote 3	70 m	0.7 m
Mote 4	100 m	1.05 m
Mote 5	130 m	1.5 m

**Table 2.** Predicted Probabilities Revealing Actual Situations on Sensor One by the ESA.

Time steps	Experiment-1	Experiment-2	Experiment-3	Experiment-4

*t*_0_	75.12%	92.80%	55.02%	67.90%
*t*_1_	78.07%	95.40%	80.01%	72.42%
*t*_2_	78.39%	94.64%	75.42%	53.79%
*t*_3_	78.76%	95.56%	95.72%	61.30%
*t*_4_	78.33%	95.89%	79.17%	55.70%
*t*_5_	81.11%	94.89%	31.97%	68.11%
*t*_6_	59.72%	96.02%	66.80%	72.33%
*t*_7_	55.60%	96.12%	91.19%	63.88%
*t*_8_	57.41%	96.08%	75.35%	79.23%
*t*_9_	57.12%	96.23%	74.83%	62.25%
*t*_10_	50.26%	97.07%	54.56%	63.04%
*t*_11_	57.20%	97.71%	49.06%	49.57%
*t*_12_	55.68%	97.33%	58.64%	68.43%
*t*_13_	84.71%	97.74%	95.73%	67.83%
*t*_14_	87.05%	97.82%	72.06%	51.28%
*t*_15_	85.03%	97.11%	83.78%	79.36%
*t*_16_	81.62%	97.56%	95.87%	68.08%
*t*_17_	85.39%	97.59%	97.00%	50.38%
*t*_18_	82.83%	96.38%	53.05%	68.15%
*t*_19_	81.01%	96.52%	43.29%	53.84%
*t*_20_	27.91%	63.11%	31.24%	36.31%
*t*_21_	29.84%	67.49%	35.47%	39.92%
*t*_22_	27.96%	63.14%	27.56%	34.19%
*t*_23_	28.86%	66.94%	22.00%	41.38%

**Figure**	Figure 13(a)	Figure 13(b)	Figure 14(a)	Figure 14(b)
